# Data silos undermine efforts to characterize, predict, and mitigate dementia-related missing person incidents

**DOI:** 10.1177/08404704221106156

**Published:** 2022-06-09

**Authors:** Antonio Miguel Cruz, Samantha Marshall, Christine Daum, Hector Perez, John Hirdes, Lili Liu

**Affiliations:** 1 3158 University of Alberta, Edmonton, Alberta, Canada.; 2 Glenrose Rehabilitation Hospital, Edmonton, Alberta, Canada.; 3 8430 University of Waterloo, Waterloo, Ontario, Canada.

## Abstract

It is estimated that up to 60% of people living with dementia go missing at least once during the course of their disease. Databases on missing incidents involving people living with dementia are managed in silos with minimal or incomplete data. A national strategy for the collection of data on missing incidents of people living with dementia would optimize time and resources spent on police and search and rescue and enhance chances of saving lives of those who go missing. Such a strategy would be a first step toward developing strategies to prevent future missing person incidents among this population. The objectives of this manuscript are to: (1) describe the issues and challenges related to the lack of integrated data on people living with dementia at risk of going missing, and (2) propose directions to create a national database.

## Introduction

By 2030, the number of people aged 60 years and older will increase by 34% globally,^1^ and by 2040, older adults are projected to comprise one-fourth of the overall.^2^ Dementia is an age-related chronic disease associated with disability and dependency.^3^ In Canada, the number of people living with dementia is rising, with prevalence rates doubling every 5 years for Canadians aged 65 years and older.^4^

Dementia is associated with long-term care placements, substantial challenges for family caregivers,^5^ and increased risk of missing person incidents.^4^ It is estimated that approximately 40-60% of people living with dementia go missing at least once during the course of their disease^6^ and at least 5% become lost repeatedly.^7^

When a dementia-related missing person incident is reported, police organizations conduct several tasks before activating an official search and rescue procedure. Police personnel visit the family or caregivers to gather information and rule out a crime. If a search is warranted, a police service may use social media to share details about the missing person, with permission of the family, and conduct a search. A police service may also engage an external search and rescue team to assist.

Search strategies used for people with dementia can differ from those used for other populations as people with dementia typically continue to “go (walk) until they get stuck.”^8^ Search personnel who are not aware of behaviours associated with dementia may use traditional search and rescue methods of police vehicle dispatch and helicopters, instead of ground personnel, possibly incurring unnecessary expenses. Other strategies that leverage traditional search and rescue procedures for missing people who have dementia include the use of locating technologies, community support, and vulnerable person registries. A registry is an organized system that collects uniform data about a population based on disease, condition, or exposure and that serves one or more predetermined scientific, clinical, or policy purposes.^9^ A registry database is a file or group of files derived from the registry itself.

The objectives of this manuscript are to: (1) describe the issues and challenges related to the lack of integrated data on people living with dementia at risk of going missing, and (2) propose directions to create a national database.

## Challenges related to current siloed approaches for collecting data on missing persons

An accurate database gives a baseline of older adults who go missing, provides the frequency within a specified period of time, and would guide policy makers on strategies to address this public health concern. A national database requires consistency in what data to collect and how data are collected at local levels. Collaboration is necessary between services that contribute to the database; these sectors include healthcare, police and search and rescue teams, and other social programs. Although we are in an era of big data and artificial intelligence, we continue to collect data in silos. Barriers to data integration can be attributed to fragmentation, existing legislation on the privacy and security of health data, data ownership, and inconsistent adoption of common terminologies, data collection approaches, and data sharing practices.^10-12^ There are data silos in Electronic Health Records^12^ and in information and communication industry corporate data.^13^ Specific to missing people living with dementia, challenges include: (a) different record management systems used by organizations, some of which cannot be integrated with other systems; (b) privacy legislation that prevents collection of specific data and integration or sharing of such data; (c) data collection approaches that are not standardized nor consistent, making them time and labour intensive to manage, clean, and analyze; and (d) incomplete and missing data, as well as under-reporting of missing person incidents.

### Data silos

Incidence, prevalence, and calculation of risks depend on consistent and complete data collection. This is not possible when data are collected in silos across different services and jurisdictions. Without consistent and complete data, program planners and funders do not have adequate data to inform decision-making, or to monitor the impact of prevention and management programs. Incomplete data systems also prevent effective search and rescue operations that rely on information to understand the behavioural patterns of missing persons. Silos result from data being retained in isolation in local jurisdictions such as police services, volunteer search and research services, and local or national registries for vulnerable older adults. Data are typically collected using bespoke databases and are not shared between organizations, services, and facilities. As the databases are not standardized, the data cannot be integrated with other systems. One solution is to use unique “serial numbers” or “universal citizen identifiers” to link datasets between organizations.^14^ However, no agreement to encourage the use of such serial numbers or universal citizen identifiers is in place in Canada.

### Legislation and associated barriers for data collection, integration, and sharing

Legislation in Canada on data privacy and security has hindered efforts to create a strategy to collect data at a national level for dementia-related missing person incidents. According to the national Personal Information Protection and Electronic Documents Act^15^ and provincial and territorial privacy laws, personal data such as one’s home address and health data such as dementia cannot be disclosed or shared among or between law enforcement services and other for-profit and not-for-profit organizations without consent. Such consent is challenging to obtain from people living with dementia and their guardians.^16^ On the other hand, standardized clinical assessment databases in home care and long-term care settings provide rich and informative data about persons at risk of going missing.^17,18^ However, these data are not typically shared outside the immediate healthcare team.

### Inconsistencies in terminology and data collection practices

The inconsistent adoption of terminologies and data collection practices also hinders a national approach for data collection. There are several definitions and scenarios to describe being “lost”^19^ or “missing” which are typically not reported to the authorities if an individual is first found by the family. The term “wandering”^20^ is sometimes differentiated from “critical wandering” which is wandering resulting in a person getting lost or “eloping” which describes behaviour to leave a situation possibly to seek safety.^21^

In Canada, two initiatives use databases of person living with dementia based on registration: (1) the MedicAlert® Connect Protect Program®,^22^ an initiative that gives first responders 24/7 access to MedicAlert® subscribers’ data when they go missing; and (2) Project Lifesaver, a program implemented in some jurisdictions, aiming to protect those individuals at risk of wander and get lost. As these programs are based on registration, the databases do not include individuals who do not register. Fees may be a barrier to accessing this program. Further, access is currently limited to those who live within the service areas.

### Incomplete data and under-reporting of missing person incidents

Health data about persons at risk of going missing can explain the causes of incidents and can be used to prevent future missing incidents. Information on a missing person’s cognitive status, associated medical conditions, treatment or medications, and behaviour under stress are types of data that can inform the management of risks of going missing. However, health data is often not associated with missing incidents.

Another challenge is under-reporting of missing person incidents. Police and third-party organization data, such as MedicAlert®, do not record missing incidents when family members do not make a report, or the process to do so at a community level does not exist.^23^ This leads to under-reporting of individuals who become lost repeatedly. Thus, information kept in these databases can be incomplete, unreliable, inconsistent, and outdated.^24^

## Toward a national, coordinated approach for data on missing persons

A national, coordinated approach for data on missing persons is needed. First, registries with data about missing person incidents, including health conditions, frequency and timing of prior incidents, and outcomes would inform service providers on the likelihood of future missing incidents. An individual who has been missing several times over the past month is at higher risk of a repeated incident than another individual who went missing 5 years ago while experiencing confusion related to an infection. Second, a coordinated approach would provide consistent data to police and search and rescue personnel on ways to characterize the behaviours of lost people living with dementia. This characterization could inform personnel on specific aspects of the conditions used to prioritize resource allocation to find a missing person. First responders have reported persons with dementia can walk for long distances and even take public transit.^25,26^ Third, for people living with dementia, care partners, and dementia advocacy organizations such as the Alzheimer societies, a coordinated approach to data collection and reporting would complete our understanding of dementia-related missing person incidents and contribute to the development of guidelines to prevent future incidents. Fourth, not-for-profit organizations, such as the MedicAlert® Safely Home® Program, could better fulfil their mandate of ensuring that people with Alzheimer’s or other forms of dementia are quickly returned home safely. Analyses of missing person incidents may be useful for these organizations to determine their coverage policies or membership fees. For example, an analysis using registry data may help determine whether people living with dementia in low-income families are at the greatest risk of getting lost and, as a result, organizations may consider alternate payment options or subsidy programs for those eligible.^27^ As most missing person incidents involving dementia occur in community settings, data collection should consider the perspectives of those living with the condition and their care partners. Finally, any new registry should integrate with health information systems that include home care, community support services, long-term care, and geriatric psychiatry settings.

The creation, implementation, and adoption of a coordinated approach for data on missing person incidents involving people living with dementia at a national level are a worthwhile endeavour. There are several examples that attest to this. The Continuing Care Reporting System (CCRS),^28^ the Home Care Reporting System (HCRS),^29^ and the newer Integrated interRAI Reporting System (IRRS)^30^ databases at the Canadian Institute for Health Information (CIHI) are examples that data integration is possible. These national databases collect clinical assessment information gathered at the point of care based on the interRAI standard, including demographic, clinical, functional, and resource utilization measures on individuals receiving services across the continuum of care.^28,29,31-33^ Jurisdictions are provided with a standardized framework to track health service organization data, which provides support for planning, decision-making, and management of operations at a local level.^28,29^ Despite differences in how data are collected between jurisdictions, once data are submitted to CIHI, data transformation occurs to generate information that is reliable and comparable between jurisdictions and organizations at various levels.^28,29^ These data systems can be used to inform the development of a unified data collection method for people living with dementia and other cognitive impairments in Canada. Internationally, Sweden^34^ and China^35^ are informative examples of how both health and administrative datasets can be integrated.

A plausible solution is the creation of a simplified workflow between national data sources such as the CCRS, HCRS, and IRRS and a registry for dementia-related missing person incidents using a unique universal identifier for people living with dementia*.* This simplified workflow posits the idea that a registry for dementia-related missing person incidents will be an integrative tool that can facilitate the capture of a large variety of data sources (eg, interRAI data at the point of care) and transform them into a standardized format to be collected in a secure cloud storage facility. On the other hand, by using the unique universal identifier for people living with dementia, the registry for dementia-related missing person incidents would then be allowed to communicate with healthcare data holdings such as those based on the interRAI standard. While a coordinated strategy at a national level would be ideal, the data need to be collected locally where those responsible for these tasks are typically overwhelmed with competing priorities and have limited human and financial resources. Such an initiative would depend on agreement at local levels that a coordinated strategy for data collection would benefit people living with dementia, their care partners, communities, and service providers, including search and rescue personnel and other first responders. A coordinated strategy requires buy-in to the idea of adhering to a common, national standard and sharing data, and recognition that privacy laws and regulations may need to be amended or their interpretation clarified.

From a legislative perspective, buy-in would require assurance that the data are based on de-identified measures so that local organizations would want to participate in sharing dementia-related missing person incidents and digital health data for research, evaluation, and planning purposes. At the same time, people living with dementia and their care partners should have a say regarding who by and under what circumstances their data may be used. Stakeholders such as healthcare providers and first responders should be informed on how the data would be handled and that it would be submitted to a national organization.

The creation of a registry for dementia-related missing person incidents involves certain considerations. Standardized measurements, specifications regarding data standards and coding rules, training, reporting standards, cross-sector consistency, data quality mechanisms, and proper data architecture and technical infrastructure all have to be in place.^32,36^ In order to facilitate data analytics and data integrity processes, it would be necessary to re-code existing data to a common data model.^37^ The data systems should be structured to be compatible with global health data architecture, such as HL7^®^ (Health Level 7) and FHIR^®^ (Fast Healthcare Interoperability Resources). Also, modern information technology architecture and global or Canadian standards for data terminology and exchange should be implemented.^37^ Ideally, the system in place is interoperable with other data systems to avoid unnecessary redundancies. In simple terms, a registry of dementia-related missing person incidents could be presented in a software-as-a-service or middleware model, interacting as the presentation layer on the front end, and the registry database on the other.^9^

Best practices for the creation of local registries include: (a) defining the purposes of the registry; (b) determining whether a registry is an appropriate strategy to achieve those purposes; (c) identifying the stakeholders; (d) defining the scope, core or minimal dataset, and target population; (e) assessing the feasibility of a registry and securing funding for its maintenance; (f) implementing appropriate consent procedures; (g) implementing the mechanisms to guarantee timely and up-to-date information; and (h) adequate clinical information to be useful in formulating a response to an incident.^9^ Such registries would involve consistent use of terminologies and datasets which would feed into a national database anonymously to allow analyses of incidence, prevalence, and trends. These analyses would be shared with local jurisdictions to inform decision makers.

Health leaders could advocate for the above practices and facilitate the data integration of dementia-related missing person incidents in Canada. Registries could integrate data from various sources, including primary and secondary data. As secondary data may have been collected for purposes other than the registry and not for research purposes, they may not be equally structured compared to a registry’s primary data. As a result, identifiers are necessary to guarantee an accurate “link” and match between primary and secondary data sources. As medical record systems are potential secondary sources of data linked to registries, health leaders could facilitate the process of interfacing registries with electronic health records. Such approaches would take into ethical and legal considerations that apply to the original compilation of each dataset.

With the adoption and use of a registry for dementia-related missing person incidents, the ethical matters related to the use of data need to be considered. Studies have examined the ethical considerations related to the use of electronic tracking devices with people living with dementia.^38,39^ This literature emphasizes the importance of balancing the risks to the privacy and autonomy of a person with dementia when using tracking devices intended to provide peach of mind to caregivers. Dementia is associated with continuous change in cognitive function, and therefore requires ongoing monitor of capacity and consent. Assent to the use of these devices should be sought even where legal capacity to provide consent is not present. These ethical concerns are also relevant to the sharing of data from healthcare databases with first responders who are charged with finding a lost person with dementia. For example, a person’s loss of privacy from sharing health information should be conditional on consent. The ethical principle of beneficence applied to electronic tracking devices suggests that the benefits to a person include increased safety and security as well as reduced response times and successful search efforts for a missing person. These benefits also apply to data sharing that would improve the ability of first responders to come to the aid of a lost person with dementia in a manner that is respectful and person-centred.

There are also practical considerations related to health data sharing that have both legal and ethical implications. Specifically, in some jurisdictions, it is permissible for police and other first responders to share information about persons brought to healthcare organizations. However, it may not be permissible under privacy legislation for healthcare organizations to provide person-level clinical data to search personnel without consent when a resident goes missing.

The creation of a registry for dementia-related missing person incidents can be considered a monitoring system. Thus, we suggest health leaders examine whether such a system foster ageing in place for people who have dementia.^40^ Ultimately, a person living with dementia has the right to make informed decisions about the whether or not to be part of a registry designed to mitigate risks of missing person incidents.^41^

## Conclusion

Advances in computing power and analytics provide a unique opportunity to launch a national dialogue on the key issues relating to registry design, data sharing, and data governance on missing person incidents involving people living with dementia. Stakeholders including police, search and rescue personnel, communities, people living with dementia, and their care partners have a stake in advocating for the integration of dementia-related missing person incidents with digital health data. An integrated approach to data collection would provide a fuller understanding of missing person incidents, thereby allowing the implementation of proactive strategies.
